# *Comparison of Artemisia annua* Bioactivities between Traditional Medicine and Chemical Extracts

**DOI:** 10.2174/157340720904140404151439

**Published:** 2013-12

**Authors:** Ahmed Nageeb, Azza Al-Tawashi, Abdul-Hamid Mohammad Emwas, Zeyad Abdel-Halim Al-Talla, Nahla Al-Rifai

**Affiliations:** 1 Division of Chemical Life Sciences and Engineering, King Abdullah University of Science and Technology (KAUST);; 2 Research and Development, Qatar Foundation;; 3 NMR Core Lab, King Abdullah University of Science and Technology (KAUST);; 4 Analytical Core Lab, King Abdullah University of Science and Technology (KAUST);; 5 Department of Environmental Technology Management, College for Women, Kuwait University, Kuwait

**Keywords:** Artemisia, bioactivities, NMR, antibacterial, anticancer.

## Abstract

The present work investigates the efficacy of using *Artemisia annua* in traditional medicine in comparison with chemical extracts of its bioactive molecules. In addition, the effects of location (Egypt and Jericho) on the bioactivities of the plant were investigated. The results showed that water extracts of *Artemisia annua* from Jericho have stronger antibacterial activities than organic solvent extracts. In contrast, water and organic solvent extracts of the *Artemisia annua* from Egypt do not have anti-bacterial activity. Furthermore, while the methanol extract of EA displayed high anticancer affects, the water extract of Egypt and the extracts of Jericho did not show significant anticancer activity. Finally, the results showed that the methanol and water extracts of Jericho had the highest antioxidant activity, while the extracts of Egypt had none. The current results validate the scientific bases for the use of *Artemisia annua* in traditional medicine. In addition, our results suggest that the collection location of the *Artemisia annua* has an effect on its chemical composition and bioactivities.

## INTRODUCTION

Plant extracts are a valuable source of new therapeutically relevant natural products. *Artemisia annua* (Family-*Asteraceae*) has been used in traditional medicine for treating fever and malaria. There are several species of Artemisia known as aromatic fragrance plants that have a characteristic scent and taste [1]. Some Artemisia species are used medically because they repel helminthes of intestines. For example, in folk medicine, for the treatment of worms, it is consumed as a type of tea for three consecutive days before going to sleep [2]. In addition, other Artemisia species are recommended for neurological disorders [3, 4]. Some kinds of Artemisia are used in Iraqi folk medicine for the treatment of diabetes mellitus [5, 6]. Furthermore, a tea made from both leaves and flowers of Artemisia that grow in Egypt is used as repellent for intestinal gases and a gusher of menstruation [7]. While a certain Artemisia species that grows in Saudi Arabia is used as a cure against rheumatism and to treat cold [8], another Artemisia species in Tunisia is used in traditional medicine as decoction for their antivenin, anti-inflammatory, anti-rheumatic and antimicrobial properties [9, 10]. It has been reported that Artemisia species that grow wild in the uncultivated land in north India, and distributed from central Europe to Western Asia and Western Himalayas have medicinal properties like anti-cholesterolemic, antipyretic, antiseptic and used in the treatment of hepatitis, jaundice, and gall bladder inflammation [11]. The essential oil has strong insecticidal activity against stored product insects [12, 13]. The oil also has bio-herbicidal properties as it causes severe phytotoxicity and interferes with the growth and physiological processes of some weed species [14]. Artemisia should be taken carefully due to the santonin present, which has toxic effects if it is taken irregularly [15, 16].

Many researchers were interested in testing the antibacterial and antimicrobial activities for several types of *Artemisia annua*. Several reports show that, the chemical compositions of Artemisia differ by location and seasons of growth. Recently, Yun *et al*. 2008 tested the influence of growth season on antimicrobial and antioxidative activities of extracts from *Artemisia princeos* var. orientalis [17]. Liu *et al*., 2010 investigated the antiplasmodial activity of various extracts of Artemisia afra and annua to identify polar metabolites and metabolic differences between these species [18]. In addition, *Artemisia annua *is a well-known antimalarial herb [19]. Ozguven *et al*., (2008) determined yield and yield components, essential oil contents, and Artemisinine content of *Artemisia annua*, grown under four nitrogen applications in Turkey [20]. They found that Artemisinine content was significantly affected by nitrogen applications. Another study completed by Ma *et al*., (2007) observed *Artemisia annua *l volatile oil and found that three hundred and three components were tentatively identified with terpene compounds being the main components of this species [21]. Brown *et al*., (2003) found that the seeds are a concentrated source of almost all the secondary metabolites, as well as several novel terpenoids in which they determined fourteen sesquiterpenes, three monoterpenes and one diterpene natural product from the seeds of *Artemisia annua *[22]. Zhang *et al*. (2008) proved that *Artemisia annua* showed the strongest biological activity in July, and found that the acaricidal activity varied significantly with the development of the individual plant [23]. Most of the antibacterial studies focused on the organic extract and used only one plant source. In this study, we compared the organic solvent extracts with water extract of the *Artemisia annua *collected from different sources (Egypt and Jericho) that have been typically used in traditional medicine. Until now a comparison between the chemical composition and bioactivities of JA and EA has not been reported. 

Nuclear Magnetic Resonance (NMR) spectroscopy is a powerful analytical tool used to study the chemical composition of the extracts of a given sample [24-27]. NMR has been used intensively to search for drug candidates from natural products and for drug assessments [28], drug discovery [29, 30], and structure-based drug design [31]. In this study, we employed ^1^H NMR spectroscopy to compare the metabolite fingerprinting of Artemisia collected from Jericho and Egypt.

The main aim of this study was to investigate the potential anti-cancer and anti-bacterial bioactivities of extracts of *Artemisia annua*. In addition, we compared the bioactivities of *Artemisia annua* from Jericho (JA) and *Artemisia annua *from Egypt (EA) using different organic extracts along with the water extracts that have been used in traditional medicine. NMR spectroscopy was used to compare the chemical composition of each extract. The NMR results explained the differences in bioactivities of the studied *Artemisia annua *extracts. 

## MATERIAL AND METHODS

***Water and chemical extracts***: A 250ml and 500ml flat-bottomed Erlenmeyer flasks were used. For evaporation of solvents using a rotary evaporator, 50, 100, 250 and 500 ml round-bottomed flasks were used. Filtration was accomplished using grade 1 Whatman filter papers with a pore size of 11 μm and durapore membrane filters with a pore size of 0.22 μm (Millipore). For filtration, ceramic Büchner funnels (Coorstek) and 500 ml filtration flasks (KIMAX) were used. For air drying and weighing the residues, 20 ml disposable scintillation vials were used. 

***Extraction of Plants:*** A 10g sample of the dried flowers and flowering tops of the leaves of *Artemisia annua *were extracted using 200 ml of absolute methanol or hexane-chloroform. Polytron homogenizer was used to crush the plant material. The extraction was carried out for five days with continuous gentle stirring to get the maximum yield. After that, extracts were sonicated for 2 hours using the ultrasound homogenizer, because sonication provides an efficient method for extracting tightly bound chemicals from solid surfaces such as plants. To remove the remaining plant material and particulate matter, the extracts were filtered in 500 ml filter flasks using 11 μm pore-sized filter papers. Following this filtration, the extracts were filtered again in other 500 ml filter flasks using 0.22 μm pore-sized membrane filters to reduce bacterial contamination of the extracts. The concentration of each crude extract was 500mg/ml as determined by drying and weighing. One ml of the extract was transferred to an empty weighed 20 ml vial. The extract was air-dried and then the vial was re-weighed.

***Preparation of Extracts***: Extracts were prepared in different concentrations of dry residues in the appropriate solvents. This was done by calculating the volumes of the crude methanol extracts that could contain the desirable weights of the dry material (depending on the known concentrations of the extracts). For each concentration of one extract, the volume corresponding to the desired weight of dry residue was transferred to a round-bottomed flask and dried using the rotary evaporator. The dry residues were then dissolved in certain volumes of the appropriate solvents that would give the desired concentrations. These solutions were sonicated using the sonication bath to help dissolve any un-dissolved matter. To control bacterial contamination in anti-cancer assays, the prepared concentrations were filtered using syringe filters with 0.22 μm pores.

## Cell Viability Assay (Alamar Blue^®^ Assay)

To test for the anticancer effects of the extracts, three cell lines were used: Human Breast Adenocarcinoma MCF7, Human Lung Carcinoma and Chinese Hamster Ovary cells. In addition, primary cells were obtained from Invitrogen™: Primary Human Dermal Fibroblasts isolated from adult skin (HDFa). The Alamar Blue^®^ reagent was used to determine the cell viability percentages. The cell count was adjusted to 10^4^ cells /ml for each cell line. Two hundred μl of cell suspension of each cell line were added to six wells of a flat-bottomed 96-well plate with a lid. The plate was incubated at 37°C in a 5% CO_2 _atmosphere for one day. After that, the growth media were aspirated from each well and solutions of tested extracts in the growth media were added to the wells. In addition, two controls were included in the plate: 70% DMSO in the growth media and positive growth controls by adding growth media to the cell-containing wells. The plate was incubated for 2 days at 37°C in a 5% CO_2_ atmosphere. After incubation, 25 μl of the indicator (Alamar Blue^®^ alamarBlue^®^ Cell Viability Assay, Life technology) were added to each well. The plate was incubated for an additional 3 hours at 37°C in a 5% CO_2 _atmosphere. After that, the plate was read using the micro plate spectrophotometer. (Absorbance at 570 nm and 600 nm wavelengths) 

Calculations were performed according to the manufacturer’s manual instructions as follow:

Since absorbance is directly proportional to the product of the molar extinction coefficient and concentration, a pair of simultaneous equations was obtained from which the two unknown concentrations could be determined:

(1) $$C_{\mathrm{RED}}\left(\varepsilon \mathrm{RED}\right)\lambda_{1}+\mathrm{COX}\left(\varepsilon \mathrm{OX}\right)\lambda_{1}=A\lambda_{1}$$

(2) $$C_{\mathrm{RED}}\left(\varepsilon \mathrm{RED}\right)\lambda_{2}+\mathrm{COX}\left(\varepsilon \mathrm{OX}\right)\lambda_{2}=A\lambda_{2}$$

To solve for the concentration of each component, we used the following two equations:

(3) $$C_{\mathrm{RED}}=\frac{\left[\left(\varepsilon \mathrm{OX}\right)\lambda_{2}A\lambda_{1}-\left(\varepsilon \mathrm{OX}\right)A\lambda_{1}A\lambda_{2}\right]}{\left[\left(\varepsilon \mathrm{RED}\right)\lambda_{1}\left(\varepsilon \mathrm{OX}\right)\lambda_{2}-\left(\varepsilon \mathrm{OX}\right)\lambda_{1}\left(\varepsilon \mathrm{RED}\right)\lambda_{2}\right]}$$

(4) $$C_{\mathrm{OX}}=\frac{\left[\left(\varepsilon \mathrm{RED}\right)\lambda_{1}A\lambda_{2}-\left(\varepsilon \mathrm{RED}\right)A\lambda_{2}A\lambda_{1}\right]}{\left[\left(\varepsilon \mathrm{RED}\right)\lambda_{1}\left(\varepsilon \mathrm{OX}\right)\lambda_{2}-\left(\varepsilon \mathrm{OX}\right)\lambda_{1}\left(\varepsilon \mathrm{RED}\right)\lambda_{2}\right]}$$

We used the following two equations to determine the percentage of reduction of Alamar Blue^®^:

(5) $$\%\mathrm{Reduced}=\frac{\left[C_{\mathrm{RED}}\mathrm{test}\;\mathrm{agent}\;\mathrm{dillution}\;\mathrm{well}\right]}{\left[C_{\mathrm{OX}}\;\mathrm{Untreated}\;(\mathrm{negative})\;\mathrm{control}\;\mathrm{well}\right]}$$

(6) $$\frac{\left[\left(\varepsilon \mathrm{OX}\right)\lambda_{2}A\lambda_{1}-\left(\varepsilon \mathrm{OX}\right)\lambda_{1}A\lambda_{2}\right]}{\left[\left(\varepsilon \mathrm{RED}\right)\lambda_{1}A^{'}\lambda_{2}-\left(\varepsilon \mathrm{RED}\right)\lambda_{2}A^{'}\lambda_{1}\right]}\mathrm{x100}\%$$

To calculate the percentage of difference in reduction between treated and control cells cytotoxicity/proliferation assays, we used the following equation:

(7) $$\frac{\left[\left(\varepsilon \mathrm{OX}\right)\lambda_{2}A\lambda_{1}-\left(\varepsilon \mathrm{OX}\right)\lambda_{1}A\lambda_{2}\;\mathrm{of}\;\mathrm{test}\;\mathrm{agent}\;\mathrm{dillution}\right]}{\left[\left(\varepsilon \mathrm{RED}\right)\lambda_{2}A^{o}\lambda_{1}-\left(\varepsilon \mathrm{RED}\right)\lambda_{1}A^{o}\lambda_{2}\;\mathrm{of}\;\mathrm{untreated}\;\mathrm{control}\right]}\mathrm{x100}\%$$

(8) $$\mathrm{Cytotoxicity}(\%)=\frac{\mathrm{Abs}.\mathrm{in}\;\mathrm{wells}\;\mathrm{of}\;\mathrm{test}\;\mathrm{sub}.-\mathrm{Abs}.\;\mathrm{of}\;\mathrm{negative}\;\mathrm{control}}{\mathrm{Abs}.\;\mathrm{of}\;\mathrm{possitive}\;\mathrm{control}-\mathrm{Abs}.\;\mathrm{of}\;\mathrm{negative}\;\mathrm{control}}\mathrm{x100}\%$$

Where:

- CRED = the concentration of reduced form Alamar Blue^®^ (RED); COX = the oxidized form of Alamar Blue^®®^ (BLUE); εOX= the molar extinction coefficient of Alamar Blue^®^ oxidized form (BLUE); εRED= the molar extinction coefficient of Alamar Blue^®^ reduced form (RED); A = absorbance of test wells; A’ = absorbance of negative control well. (The negative control wells contained media and Alamar Blue^®^ but no cells); A° = absorbance of positive growth control wells; λ1 = 570 nm; λ2 = 600 nm; The εOX of Alamar Blue^®^ oxidized form at 570 nm wavelength = 80,586; The εRED of Alamar Blue^®^ reduced form at 600 nm wavelength = 117,216.

## Cytotoxicity Assay (LDH)

To test for the cytotoxic effect of the extracts, the same cell lines and primary cells previously mentioned were used. The LDH Cytotoxicity Detection Kit (Promega) was used. The kit included two reagents, solution A: a catalyst (diaphorase/NAD^+^, lyophilisate) and solution B: a dye (iodotetrazolium chloride and sodium lactate). Two hundred μl of cell suspension of each cell line were added to six wells of a flat-bottomed 96-well plate with a lid. The plate was incubated at 37 °C in a 5% CO_2_ atmosphere for one day. After that, the growth media were aspirated from all wells and different concentrations of the tested extracts in growth media were added to the wells. In addition, two controls were included in the plate, a positive killing control and a negative killing control. The positive control included 1% Triton X-100 in growth media. The negative control included growth media cell-containing wells. The plate was incubated for 2 days at 37 °C in a 5% CO_2_ atmosphere. The reaction mixture was prepared by mixing 250 μl of solution A (catalyst) with 11.25 ml of solution B (dye solution). After that, the plate was centrifuged at 250Xg for 10 minutes. Then, 100 μl of the supernatant in each well were removed carefully, without disrupting the pellet, and transferred into a corresponding well of another 96-well flat-bottomed micro titer plate. To each well in the second plate, 100 μl of the reaction mixture were added. The plate was incubated away from light at room temperature for 30 minutes. After that, the absorbance of the samples was measured at a wavelength of 492 nm. The percentage of cytotoxicity was calculated using the following equation:

Calculations were performed according to the manufacturer’s manual instructions as follow:

## Determination of Antibacterial Activity

To test for the antibacterial activity of the extracts, GC5 competent *Escherichia coli* cells were used (Invitrogen™). The bacterial cell suspension was prepared from a 24 h culture and adjusted to the desired inoculum density. A disk diffusion assay was used to determine the antibacterial activity of the extracts. Disks were obtained from grade1, 11 µm pore-sized filter papers (Whatman) using a paper puncher. Then, disks were exposed to UV radiation in a Class-II Biological Safety Cabinet for sterilization. After that, the disks were soaked in different concentrations of solutions of tested materials in 8% DMSO in water and controls for 12 hours. In addition, 100 mg/ml ampicillin solution in distilled water was prepared and disks were soaked in it to serve as positive controls. In addition, some disks were soaked in the solvent of the tested materials (8% DMSO) to serve as negative controls. Then, the inoculum was applied to new thawed agar plates and spread properly using a cell spreader. Disks that were soaked in the solutions of tested materials and the controls were applied manually to the agar plates using tweezers. The plates were left for 30 minutes to dry. Then, they were incubated for 24 hours in an inverted position at 37º C in an Isotherm Economy Lab Incubator. Following the incubation process, images of the plates were taken using a KODAK Image Station 4000 MM. The lengths of zones of inhibition were measured using a ruler. 

## Determination of Particle Scavenging Activity (Anti-Inflammation)

Particles of 1,1-diphenyl-2-picryl-hydrazyl (DPPH) (Sigma Aldrich) was used to test for the antioxidant activity of the extracts. The tested extracts were prepared in different concentrations of dry residues in absolute ethanol. DPPH particles were dissolved in absolute ethanol to give a concentration of 1 mM. To each well of a 48-well micro titer plate, 500 µl of the tested solutions and 125 µl of DPPH in ethanol were added and mixed gently. A concentration of 50 µg/ml of ascorbic acid in ethanol was used as a positive control. Negative control wells contained 500 µl of absolute ethanol and 125 µl of DPPH in ethanol. The plate was covered and incubated at 37 °C for 30 minutes in an isotherm Economy Lab Incubator. After that, the absorbance in each well was measured at a wavelength of 517 nm using a spectrophotometric micro titer plate reader. 

Calculations were performed according to the manufacturer’s manual instructions as follow:

The free radical scavenging activity was calculated using the following equation:

(9) $$\mathrm{Scavenging}\;\mathrm{Effect}(\%)=\frac{A_{0}-A_{1}}{A_{0}}\mathrm{x100}$$

Where:

- *A*_0_ is the absorbance of the negative control reaction
- *A*_1_ is the absorbance in the presence of the sample of the tested extracts

## NMR Experiments 

The NMR samples were prepared by dissolving the compounds in 1 ml of deuterated solvents (methanol CD_3_OD, D_2_O) and then 0.6 ml of the solution was transferred to 5 mm NMR tubes. NMR spectra were acquired on a 700 AVANAC III spectrometer equipped with TXI CryoProbes and a Bruker 600 AVANAC III spectrometer equipped with a BrukerBBO multinuclear probe. The ^1^H NMR spectra were recorded by collecting 128 scans with a recycle delay time of 5s. Exponential line broadening of 1 Hz was applied before Fourier Transformation. Bruker Topspin 2.1 software was used in all experiments to collect and analyze the data. 

## RESULTS

## Antibacterial Activity

**Fig. (1):**
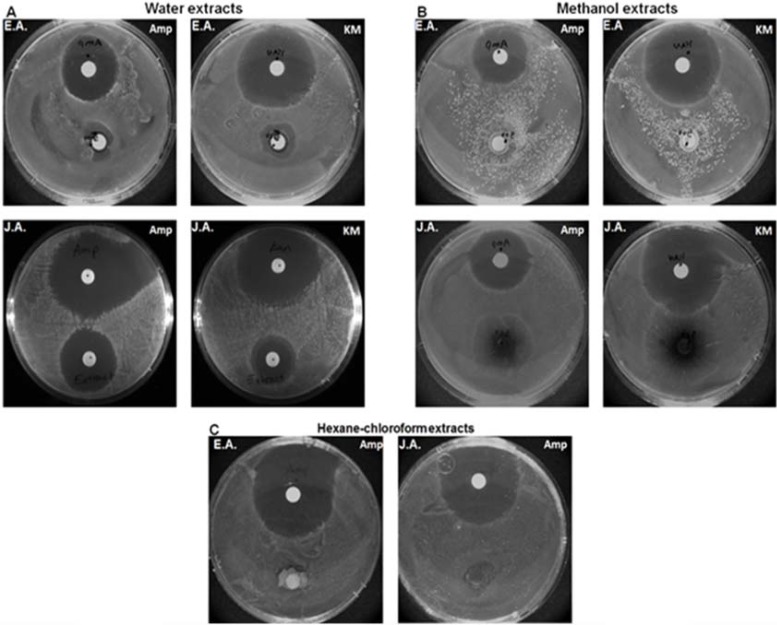
A disk diffusion assay to test the Antibacterial Activity of Egyptian Artemisia, EA and Jericho Artemisia, JA extracts. Filter paper (Whatman) disks were soaked in A. EA and JA leaves’ water extracts (500mg/ml), B. EA and JA leaves’ methanol extracts (500mg/ ml), C. EA and JA leaves’ Hexane-chloroform extracts (500mg/ml), and in Ampicillin (100 mg/ml) and Kanamycin (25 ug/ ml) as positive controls for 12 hours. In all cases, disks were applied separately, each extract with a positive control, to *Escherichia coli* agar plates and incubated for 24 hours at 37C°.

The antibacterial activity of *Artemisia annua *(both from the Egyptian Artemisia, EA and the Jericho Artemisia, JA) leaf extracts was examined in comparison with *Escherichia coli.* The extractions were carried out using hexane-chloroform, methanol and water for both plants (six extracts in total). The antibacterial activity of the extracts showed varying magnitudes of inhibition patterns with the standard positive control. Out of the six extracts tested, the JA water extract showed the most significant antibacterial activity (Fig. **1A**). In addition, the JA methanol extract showed higher antibacterial activity in comparison to the EA methanol extract while the hexane-chloroform extracts of both plants showed no antibacterial activity (Figs. **1B** and **1C**). 

## Anticancer Activity

**Fig. (2):**
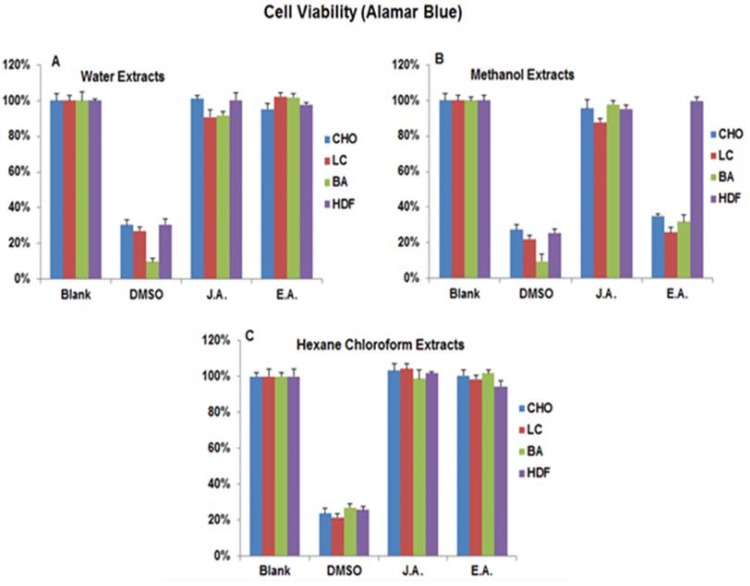
Cell viability assay to test the Anti-cancer Activity of Egyptian Artemisia, EA and Jericho Artemisia, JA extracts. Human Breast Adenocarcinoma MCF7 (BA), Human Lung Carcinoma (LC) and Chinese Hamster Ovary (CHO) cell lines and Primary Human Dermal Fibroblasts isolated from adult skin (HDFa) cells were used. 104 cells /ml of each cell type was incubated separately with A. EA and JA leaves’ water extracts (500mg/ml), B. EA and JA leaves’ methanol extracts (500mg/ ml), C. EA and JA leaves’ hexane-chloroform extracts (500mg/ml), and 70% DMSO in the growth media as positive control. Alamar Blue® reagent was used to determine the cell viability percentages. Readings were taken using the micro plate spectrophotometer (Absorbance at 570 nm and 600 nm wavelengths).

By using the Breast Adenocarcinoma (BA), Chinese Hamster Ovary (CHO), Lung Carcinoma (LC) cancer cell lines and the Human Dermal Fibroblast primary cells from adults (HDFa), we tested the anticancer activity of the hexane-chloroform, methanol and water extracts of both JA and EA Briefly, cells were plated in 96-well plates for 24 hours, treated with each extract separately and then we used the Alamar Blue^®^ (according to the manufacturer’s instructions) as a cell viability detector. Intriguingly, out of the six extracts, the EA methanol extract had the highest anti-cancer activity (Fig. **2B**). In comparison to the untreated cells (negative control) and the DMSO treated cells (Positive control), the EA methanol extract killed three times more BA and LC cancer cells and almost two times more CHO cancer cells while it did not have much effect on the HDFa primary cells. In addition, neither the EA water and hexane-chloroform extracts nor the JA extracts exhibited significant anticancer activity (Fig. **2A** and **C**).

**Fig. (3):**
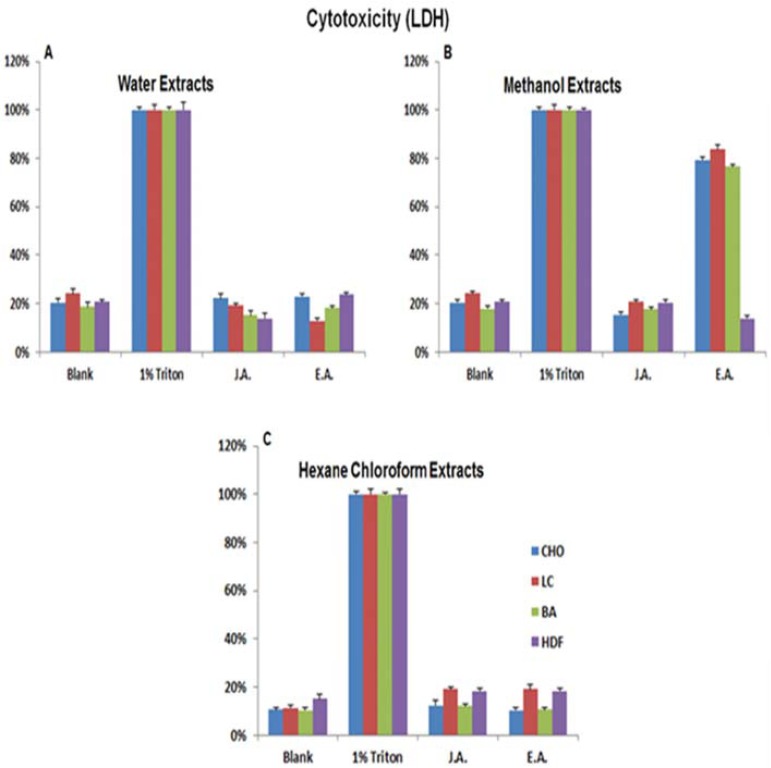
Cytotoxicity Assay (LDH) to test the Anti-cancer Activity of Egyptian Artemisia, EA and Jericho Artemisia, JA extracts. Human Breast Adenocarcinoma MCF7 (BA), Human Lung Carcinoma (LC) and Chinese Hamster Ovary (CHO) cell lines and Primary Human Dermal Fibroblasts isolated from adult skin (HDFa) cells were used. 104 cells /ml of each cell type was incubated separately with A. EA and JA leaves’ water extracts (500mg/ml), B. EA and JA leaves’ methanol extracts (500mg/ ml), C. EA and JA leaves’ Hexane-chloroform extracts (500mg/ml), and 1% Triton X-100 in growth media as positive control. LDH Cytotoxicity Detection Kit was used to determine the cell viability percentages. Readings were taken using the micro plate spectrophotometer (Absorbance at 492 nm wavelengths).

To confirm our results, we retested for the cytotoxic effect of the extracts on the same cell lines and primary cells used in the Alamar Blue^®^ experiment using an LDH kit according to the manufacturer’s instructions. In agreement with the Alamar Blue^®^ results, the EA methanol extract exhibited the highest cytotoxicity effect (Fig. **3B**). It showed 100% cytotoxicity to LC and CHO cells and about 50% cytotoxicity to BA cells while having no effect on the HDFa primary cells. On the other hand, neither the EA water and hexane-chloroform extracts nor the JA extracts exhibited significant anticancer/ cytotoxic activity (Fig. **3A** and **C**). 

## Antioxidant Activity (Anti-Inflammation)

**Fig. (4):**
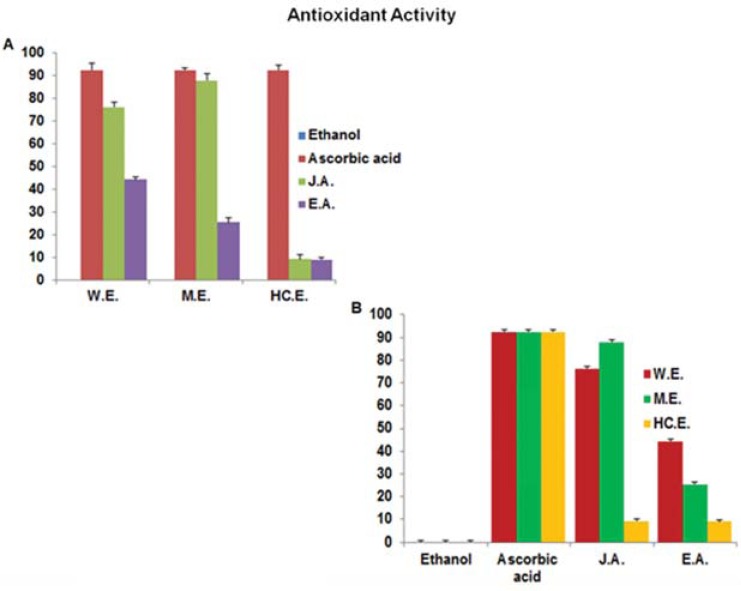
Antioxidant Activity to test the Anti-Inflammation Activity of Egyptian Artemisia, EA and Jericho Artemisia, JA extracts. Dry residues of EA and JA leaves’ water, methanol and hexane-chloroform extracts were resolved in absolute ethanol to a final concentration of (500mg/ml). A concentration of 50 µg/ml of ascorbic acid in absolute ethanol was used as a positive control. Absolute ethanol was used as negative control. Particles of 1, 1-diphenyl-2-picryl-hydrazyl (DPPH) was used to test for the antioxidant activity of the extracts. Readings were taken using the micro plate spectrophotometer (Absorbance at 517 nm wavelengths). Results were blotted according to A. Type of extract, B. Type of plant and controls.

Using 1,1-diphenyl-2-picryl-hydrazyl (DPPH), we examined the antioxidant activity of the extracts. According to our results, in comparison to the absolute ethanol sample (negative control) and the ascorbic acid sample (positive control), the JA methanol and water extracts had the highest antioxidant activity while the hexane-chloroform extract had none. On the other hand, while the EA water extract displayed about 50% antioxidant activity, its methanol extract showed a weak antioxidant activity, and its hexane-chloroform extract displayed none (Figs. **4A** and **B**).

## NMR Results

**Fig. (5):**
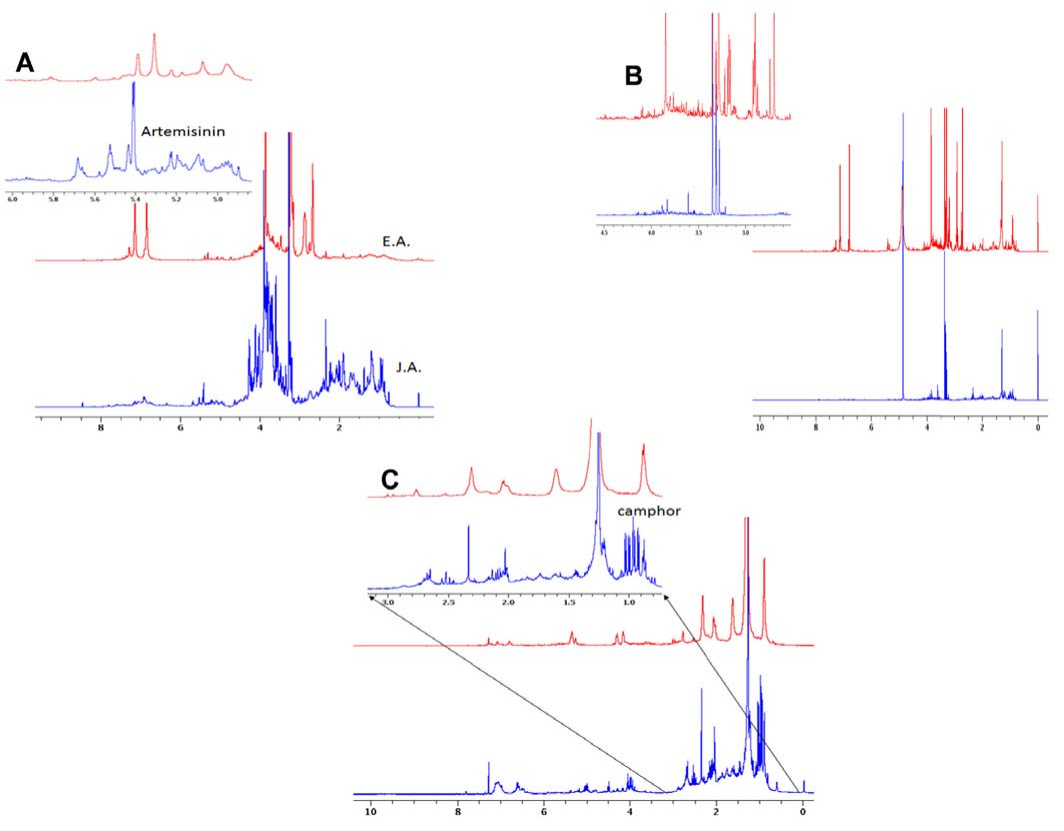
NMR analyses of the chemical compositions of the Hexane-chloroform, Methanol and Water extracts of both JA and EA A. The NMR spectrum of the JA and EA water extracts. B. The NMR spectrum of the JA and EA Hexane-chloroform extracts. C. The NMR spectrum of the JA and EA Methanol extracts.

The chemical compositions of the hexane-chloroform, methanol and water extracts of both JA and EA (six extracts in total) were analyzed by NMR. The ^1^H NMR spectra of the water extracts show clear differences between the chemical compositions of JA and EA The JA spectra show strong peaks around 0.9 ppm in comparison to a weak broad peak in the EA spectrum. These peaks were assigned to be related to camphor [32]. The spectrum of the JA water extract had a few resonances around 5.6 ppm with higher intensities than the corresponding peaks in the EA spectrum (Fig. **5A**). On the other hand, the spectra of the organic extracts (Fig. **5C**) revealed the main difference between the EA and JA The proton NMR spectrum of EA had a few broad peaks mainly in the CH3 and CH2 region with a similar spectral signature as fatty acids, confirming high level of oil concentration. Unlike the EA spectrum, the JA proton spectrum had more sharp peaks, especially around the 0.9 ppm region. In addition, the NMR spectrum of the EA methanol extract exhibited more signals than the corresponding one of the JA extract (Fig. **5B**). In comparison to the JA methanol extract, the proton NMR results of the EA methanol extract showed strong signals at (1.279, 2.694, 2.893, 3.279, 3.346, 3.846, 6.764, 7.100 ppm), indicating a high concentration of one or more metabolites that dominate the NMR spectra. These extra lines may explain the anticancer activity of EA methanol extract compared with the JA extract. In summary, the NMR results provide an explanation for the extra bioactivities of JA samples compared to associated EA samples.

## DISCUSSION

The differences between the bioactivities of the water extracts of *Artemisia annua *(used in traditional medicine) and chemical extracts (methanol and hexane-chloroform) have been evaluated for JA. and EA. The results showed that the water extracts have stronger antibacterial properties. This validates its long use in traditional medicine made from water extracts of Artemisia for treatment of malaria and other bacterial infections. We also examined the differences in the activities between *Artemisia annua *’ s samples collected from two different areas. Intriguingly, our results showed that the same *Artemisia annua *species collected from different areas exhibited different activities. These results indicate that environmental factors play a role in formulating the plants, resulting in significant differences in bio-activities. Our results may be explained by the higher temperature and dry weather in Jericho where the JA extract displayed significantly high antioxidant and antibacterial activities than the EA one. On the other hand, while both of JA and EA traditional medicine extracts showed no anticancer activity, the EA methanol extract exhibited significant anticancer activity, which was not reported before this study. These results can be utilized for further investigation to determine the anti-cancer bioactive compounds and possible use of these compounds in treating cancer.
